# The management of asthmatic smokers

**DOI:** 10.1186/s40733-016-0025-7

**Published:** 2016-06-20

**Authors:** José Miguel Chatkin, Cynthia Rocha Dullius

**Affiliations:** Faculdade de Medicina PUCRS; Hospital Sao Lucas PUCRS; Centro Clínico, Av. Ipiranga 6690, Office 501, Porto Alegre, RS CEP 90610-000 Brazil

**Keywords:** Asthma, Smoking, Smoking cessation, Anti asthmatic agents

## Abstract

Asthma is still a major public health problem in most countries; new strategies to better control this disease are necessary. Such strategies must include predisposing factors. One of these factors is smoking and a significant fraction of asthmatics are smokers. However, clinical trials studying new drugs or newer therapeutic regimens for asthma generally exclude smokers. Therefore, there is a lack of specific information about the treatment of asthma in smokers. The asthmatic smoker is a special phenotype with important therapeutic and prognostic clinical implications. Any form of tobacco use, especially cigarette smoking, plays an important role in this disease. Asthmatic smokers are prone to several negative outcomes. Smoking cessation results in an improvement of symptoms and pulmonary functioning. Counselling and first-line medications for smoking cessation (nicotine replacement therapy, bupropion and varenicline) significantly increase quitting rates. The role of electronic cigarettes in this group of patients has only begun to be studied. The treatment of asthmatics that smoke has characteristics that need must be well understood by clinicians, especially the poor response to corticosteroids. This condition is not universal and physicians should always consider its inclusion in the treatment of these patients. The association of inhaled corticosteroids (ICS) plus a long-acting beta2 adrenegic (LABA) by smoking asthmatics results in more pronounced improvement in several asthma outcomes compared with the use of corticosteroid alone. Inhaled corticosteroids in extra-fine particles associated with LABA may be a new perspective of treatment. Also the use of leukotriene antagonists may become another therapeutic alternative. The purpose of this narrative review is to discuss the challenges faced by clinicians to control asthma in smokers and to present methods of coping with smoking treatment and avoiding relapses.

## Background

Asthma affects approximately 300 million people worldwide and is responsible for 250,000 deaths each year [1]. In addition, there is evidence that the prevalence of asthma is increasing. Asthma furthermore accounts for a significant percentage of public health budgets [1, 2].

There is accordingly a pressing need for studies to reveal strategies to better control this disease, particularly severe and difficult-to-treat cases [2].

In this perspective, smoking plays an important role since the group of asthmatic smokers has special characteristics compared with nonsmokers (e.g., increased morbidity and mortality, more severe symptoms, much more difficult asthma control, higher frequency of exacerbations, worse quality of life, and increased number of life-threatening asthma attacks [1]. Decline of lung function is accelerated among these patients [1], and the risk of permanent chronic airflow obstruction is increased [3]. Among nonatopic asthmatics, smoking triples the risk of airflow obstruction; in the atopic asthmatics group the increase is not negligible although it is not statistically significant [3].

Smoking and not asthma per se is primarily responsible for the poor prognosis in lung cancer, cardiovascular comorbidities and even death in current or ex-smokers with asthma [4].

A significant fraction of asthmatics are smokers, and the prevalence of smoking among asthma patients is very close to that of the general population, around 20 % [5]. However, there have been studies showing that the frequency of smoking among these patients may be much higher [6]; at the same time, a lower incidence of asthma among active smokers has also been described [7].

Asthmatic patients start smoking at an age similar to non-asthmatics and continue to smoke in a very similar proportion [8], but the link between smoking and new adult-onset asthma is not clear. Many cohort studies have shown such association, but this finding is not a unanimous [7, 8]. Considering these controversies, the 2014 US Surgeon General’s Report on the Health Effects of Smoking states that meanwhile a biologically plausible relationship exists between active smoking and new-onset asthma, the evidence of the link between active smoking and the incidence of new-onset asthma in children, adolescents and adults ‘is suggestive, but not sufficient to infer a casual association’. However, the evidence is sufficient to infer a causal relationship between active smoking and poor asthma control and exacerbations in adults, but not in adolescents [9].

Aanerud et al [10] studied over than 15,000 persons and detected that asthma among never-smoking individuals was rare (0.4 %); the disease was more prevalent among ex- or current smokers (>12 %). In early-onset asthma, the risk of airway obstruction severely increases, regardless the smoking status of the patient. However, these authors found that among smokers with late-onset asthma, the risk of airway obstruction compared with never-smokers was higher. Recently, Coogan et al [11] reported in a longitudinal study with more than 14 years of follow up that both active and passive smokers exhibited an increased incidence of new adult-onset asthma (approximately 40 % and 20 %, respectively) compared with non-smoking asthmatics. There was also a dose-response relationship between the incidence of asthma and the higher number of pack-years smoked.

However, clinical trials studying new drugs or therapeutic regimens for asthma generally exclude smokers [8, 12]. More recently, this concern was also extended to former smokers [13–15]. The rationale for such exclusion is that tobacco use by asthma patients is associated with numerous adverse outcomes, making it difficult to analyze the real effectiveness of the drug being tested [16].

The problematic differential diagnosis between asthma and Chronic Obstructive Pulmonary Disease (COPD) is another issue that requires better understanding. This subject may introduce significant limitations in studies that include patients’ self-report of the diagnosis without performing pulmonary function tests or having confirmed medical diagnosis. The term Asthma-COPD Overlap Syndrome (ACOS) is being introduced and used for smoking patients with reversible airflow obstruction plus eosinophilia and atopy or a history of past asthma; that is, they share features of asthma and COPD [3]. Smoking adversely affects lung function of asthmatics, adding another disease (COPD) or worsening pre-existing asthma. However, the mechanisms of how smoking affects pre-existing asthma are not fully understood [4].

All these items complicate the assessment of new drugs by mixing different phenotypes and different diseases [17–24].

Therefore, there is a lack of specific information about the treatment of asthma in smokers and there is not strong evidence about the effectiveness of each drug. The treatment of smoking asthmatics has special characteristics as the poorer response to corticosteroids compared with nonsmokers [4, 8, 23–27]. Also, there is a need for more information about smoking treatments in asthmatic patients. These aspects stress the urgent need for clinical trials designed especially to reveal the best strategies to address this very frequent association of clinical conditions [4, 8].

The predominant cellularity in most nonsmoking asthmatic phenotypes is the high percentage of eosinophils. The increase in mast cells and neutrophils detected in bronchial washings demonstrates the action of pro-inflammatory agents present in cigarette smoke on the airways of an asthmatic subject [27]. The role of smoking in the structure of the peripheral airways of asthmatic smokers is now being recognized [28, 29]. There are significant differences in the metabolism of arachidonic acid in the lipoxygenase or cyclooxygenase routes in smokers compared with never-smokers [30].

Environmental air contamination caused by cigarette smoke has deleterious effects on nonsmoking asthmatics. This contamination is associated with poorer quality of life, worse lung function, increased frequency of rescue medication and more visits to emergency rooms and hospitalizations [30].

Another form of environmental contamination, no less serious, is the intrauterine exposure of a fetus through a smoking mother [31]. Prenatal exposure is associated with an increased risk of wheezing and poorly controlled asthma in early life and in older children [32, 33]. Maternal smoking during pregnancy has a smoking dose-response relationship in the offspring with the number of asthma wheezing crises up until 15 years of age (*P* = 0.023), the presence of persistent wheezing (*P* = 0.034) and asthma-confirmed diagnoses (*P* = 0.023) [34].

In addition, a grandmother who smoked during the pregnancy of her daughter transmits an increased risk of asthma to her grandchildren, even if the second-generation mother did not smoke [35, 36]. The multigenerational epigenetic effect of nicotine on lung function has already been demonstrated [37].

The negative connection between smoking and asthma has a significant effect on the public health system, resulting in an increased use of several health professionals [1, 13, 15, 38] Furthermore, smoking cessation is associated with improved symptoms and better pulmonary function results, although there have been only few studies specifically designed to test these outcomes [8, 18, 39, 40].

The asthmatic smoker is likely a special phenotype with important clinical implications. Specific approaches are necessary, and the prognosis of these individuals impact everyday medical practice [4, 11, 27]. These patients may be included in a asthma phenotype group that is associated with environmental exposures (occupational agents, cigarette smoke, air pollution, cold dry air) [41]. There is clear evidence that smokers with asthma have a different type of inflammation compared with nonsmokers with asthma.

The purpose of this narrative review is to discuss the challenges faced by clinicians in helping asthmatic smokers to quit smoking and in better control asthma in smokers.

### Smoking cessation treatment in asthmatic patients

The management of asthmatic smokers should begin with emphatic recommendations to stop smoking, including having no contact with environmental smoke [13, 42] (Table 1).

**Table 1:** How to approach asthmatic smokers

When address the smoking problem	Strategy
During each medical visit	Encourage asthmatics to stop smoking; offer advice and information about structured programs to stop smoking.
Whenever possible	Motivate asthmatics to avoid exposure to secondary cigarette smoke
Advice for parents and caregivers	Do not allow smoking in the environments in which children live, especially in sleeping room and automobiles.
In the initial evaluation of the patient	In patients with a history of smoking > 10 pack-years, evaluate the possibility of Asthma-COPD Overlap Syndrome (ACOS)

Stopping smoking is not an easy task for every smoker, but it is particularly difficult for an asthmatic smoker [43]. Quitting rates among these patients are very low [44], since these patients typically do not recognize that they are at risk of more severe asthma. Additionally, these individuals often seem to be less interested in quitting smoking. This behavior possibly has socio-psychological explanations, such as the need to feel closer to their peers and the idea of not obeying health restrictions that could lead to any kind of discrimination [8, 27]. This attempt to not feel less physically able can also explain the lower enrollment in specific educational programs, the earlier onset of smoking and the longer history of smoking before starting the process of smoking cessation [43]. Differences in smoking rate or the degree of dependence may be responsible for the increased difficulties that smokers with asthma face when quitting smoking, although the risk factors for failure in the treatment of smoking in asthma have not yet been fully studied [27, 45]. Depression, low socio-economic background and poor educational achievements are more frequent in asthmatic smoker and probably play a role in their poorer success rates [44].

Reversal of asthma symptoms and improvement in lung function and in various other outcomes occur in asthmatic smokers who stop smoking, confirming that smoking cessation has a positive result on this group of asthmatics. However, there are not sufficient data about the reversibility of the airway inflammation after a long period of smoking abstinence [18, 19, 27, 39, 40, 46–48].

In a prospective study, Tonnensen et al. [40] published that asthmatic smokers who stopped smoking experienced a significant improvement in quality of life, decreased hyperreactivity and a reduction in their usage of rescue medications. Among patients who only reduced their tobacco load, the results were less striking, suggesting a possible dose-response related to the number of smoked cigarettes. In 2006 Chaudury et al. [18] showed that asthmatic ex-smokers exhibited significant improvement in FEV1, asthma control, recovery to corticosteroid response and decrease in neutrophil counts in sputum compared with individuals who continued to smoke. Piccillo et al. [39] in 2008 reported maintained improvement in airway hyperresponsiveness (AHR) one year after smoking cessation, which was not found in their control group.

A plausible explanation for the improvement in various outcomes is that there is a gradual and progressive reduction of pro-inflammatory effects in the airways related to the decreased inhalation of cigarette smoke [39, 47].

Clinicians must emphatically inform asthmatic patients about the additional risks of smoking and the positive aspects related to quit smoking. They also have the responsibility to help patients quit smoking and explain to them that numerous attempts and/or a long treatment may be needed before reaching the definitive withdrawal.

The need to initiate the smoking cessation process should be clear and address the benefits of such action and the harm of continuing to smoke in a way that is specific to individual. Asthma symptoms, especially coughing, may even worsen during the first weeks of smoking cessation, and a temporary increase in asthma medications may be necessary [13, 40]. The physician should fully explain the patient about such possibility that it is related to the initial recovery of the self-clearing mechanisms of the airways.

Clinicians should be able to establish a relationship of trust with the patient and to understand the difficulties that will arise before reaching complete abstinence. Furthermore, physicians should be acquainted with behavioral techniques, beyond simple advice. They should receive training about dealing with everyday situations that generate trigger conditions for smoking, how to approach spouses, family members and close friends who smoke. These well-targeted strategies are important to prevent relapses [49].

Considering that tobacco dependence is a very difficult addiction to break and that the majority of smokers typically experience multiple periods of remission and relapse, physicians must know how to cope with the withdrawal syndrome and the consequent relapse [49]. However, it is important to assess the presence and intensity of withdrawal symptoms. Several questionnaires are useful for identifying patients that may need special care. These questionnaires include the Minnesota Nicotine Withdrawal Scale (MNWS), the Wisconsin Smoking Withdrawal Scale (WSWS) and the Cigarette Withdrawal Scale (CWS) among others [49].

The first-line medications for smoking cessation are nicotine replacement therapy, bupropion and varenicline (Table 2) [50]. The use of these drugs increases success rates when compared with stopping without medication. Varenicline offers significant improvement in the rates of long-term maintenance of tobacco abstinence [50]. Westergaard et al [51] reported recently the role of varenicline specifically among asthmatic smokers. This drug not only increased the probability of success with tobacco cessation but also resulted in significant improvements in airway hyperresponsiveness in the varenicline group; no change was observed in the placebo group. Serious adverse effects are not frequent, but clinicians must be aware that under certain conditions changes in treatment may be required [50, 52].

**Table 2:** Drugs used as aids for smoking cessation [50, 52]

Drug	Dose	Common adverse effects	Advantages	Warnings or disadvantages
Nicotine patch	>10cig/day: 21 mg; <10cig/day: 14 mg Taper to lower doses	Skin irritation; insomnia	Easy to use; steady nicotine level	Slow release; not to be used during cravings
Nicotine gum	<25cig/d: 2 mg/h; >25cig/d: 4 mg/h	Mouth irritation, heartburn	Cigarette oral substitute;	Dental damage; no food or drink 30 min before use
Nicotine lozenge	1st.cig >30 min after waking: 2 mg/h; 1st.cig <30 min after waking: 4 mg/h;	Hiccups, heartburn	User controls nicotine dose	no food or drink 30 min before use
Nicotine inhaler	Inhale as needed; 6–16 cartridges/day; 10 mg/cartridges	Mouth, throat irritation	User controls nicotine dose; cigarettes oral substitute	Frequent puffing required
Nicotine nasal spray	1–2 h: once/nostril; <40 applications/day	Nasal irritation, cough, sneezing, teary eyes	Most rapid nicotine delivery	Local irritation
Bupropion SR	3 days: 150 mg/day; then 300 mg/day; start 1 wk before quit day	Insomnia; dry mouth	Oral agent; reduce weight gain	Seizures risk; psychiatric effects
Varenicline	3 days:0.5 mg/day; 4 days: 1 mg/day; then 2 mg/day	Nausea, insomnia, abnormal dreams	Dual action: relieves withdrawal and blocks reward from smoking	Psychiatric effects

*Cig/d* cigarettes per day, *mg/d* milligrams per day, *mg/h* millligrams per hour

In general, the best strategy for preventing relapse and producing high long-term abstinence rates appears to be the combined use of the most effective cessation medication available together with relatively intense cessation counseling [50]. There are studies showing that some specific combination of pharmacotherapies may be more effective than one drug alone. For instance, the combination of two NRT products, as patches, a long-acting slow-onset product, or a rapid delivery short-acting product as lozenges, gums, inhalers, or nasal sprays may result in higher quitting rates [52, 53]. However, Baker et al [54] published that the success rates of quitting using varenicline, nicotine patch, or a combination of the patch and lozenges did not differ significantly at either six months or a year of follow up. At six months, the quit rates were 23 % for the patch, 24 % for varenicline and 27 % for the combination of patch and lozenges. At one year, the quit rates were 21 %, 19 % and 20 %, respectively. The patients’ motivation to quit, provision of counseling sessions and the fact that they were not heavy smokers probably influenced their success, regardless of the used medication.

Another strategy is to combine drugs with different mechanisms of action, as in hypertension or diabetes mellitus [52].

Increasing the varenicline dose seems to be tolerable for patients but failed to increase tobacco abstinence rates. Additional analyses showed no relationship between a larger dosage of varenicline and increased success at quitting smoking [52].

In conclusion, asthmatic smokers are a special group of patients that have more difficulty achieving success in terms of quitting smoking, although it seems that they do not have higher nicotine addiction rates or higher smoking rates than smokers without asthma [43]. As a result, asthmatic smokers should receive specific medications for smoking cessation in a concurrent schema of behavioral techniques [49, 50].

New devices generically called electronic nicotine delivery systems (ENDS) are widely available. The emergence of these systems has prompted debate about their potential for helping smokers quit their habit. ENDS are also embroiled in concerns by their use among youth as well as concerns that youth using ENDS will transition to tobacco use. Furthermore, it appears that a majority of individuals attempting to quit smoking combustible cigarettes with e-cigarettes are not successful in completely weaning themselves off combustible tobacco, resulting in the dual use of combustible tobacco and electronic cigarettes.

The ENDS may be effective at helping smokers to become abstinent among smokers who cannot quit or do not want to. This new possible option may represent another perspective in the harm reduction strategy [49]. Determining the role of the electronic cigarettes for the asthmatic smokers is only beginning. In the only paper published about this issue, Polosa et al. [47], conducted a retrospective study of 18 asthmatic smokers and reported improvements in asthma control and in pulmonary function parameters when the volunteers changed from conventional cigarettes to electronic cigarettes. These authors also described a decrease in the hyperreactivity of the airways among subjects that stopped smoking with the use of electronic cigarettes. This strategy is considered to be a potentiallly useful alternative to the treatment of asthmatic smokers [55]. However, at this moment, the safety and efficacy of electronic cigarettes for asthmatic smokers needs further evaluation.

The clinician must realize that no single pharmacotherapy can serve as a universally successful treatment given the complex underpinnings of tobacco dependence and individuality of smokers. This statement is even more important when prescribing smoking cessation treatment to asthmatic subjects.

### Treating asthma in smokers

Inhaled β_2_adrenoreceptor agonists play a central role in the relief of asthma symptoms. However, there are no clinical studies specially designed to evaluate any interference of smoking on the efficacy of such agents [44].

The therapeutic response to corticosteroids varies markedly between individuals; up to one third of the asthmatic smokers show evidence of insensitivity to these drugs [56]. Since this condition is not universal, these drugs should be included in most asthma treatments. The reduction or complete absence of responsiveness among asthmatic smokers remains heightened in long-term treatments and is independent of the type and the formulation of the corticosteroids [8, 57]. However, when detecting this clinical situation it is necessary to consider a possible non-adherence to asthma treatment, the correct use of the devices and/or the continuous use of some form of tobacco.

A possible mechanism for the corticosteroids insensitivity or resistance may be the increased airway mucosal permeability in smokers, which could lead to increased clearance of inhaled corticosteroids (ICS) from the airways. Smokers also have decreased histone deacetylase (HDAC) activity, which is necessary for corticosteroids to suppress cytokine production [44].

The GINA Report, updated in 2015, notes that smoking as a risk factor for poor asthma control should be always treated. This document also states that ICS are the most effective treatment for asthma. Low doses of inhaled corticosteroids may be prescribed for patients not necessarily to reduce the burden of symptoms, but to reduce the risk of severe exacerbations, and for subjects at the step 1 asthma treatment [58].

Symptomatic asthmatic smokers at step 2 should be treated by a low- to medium-dose inhaled corticosteroid [59]. However, since the benefits in these patients may be impaired, a step-up in therapy may be required at an earlier stage, prescribing a medium/high dose ICS or ICS/LABA [1, 15].

Various reports have shown that the ICS + LABA combination yields better results in smoking asthmatics than simply increasing the corticosteroid dose in several outcomes, such as hyperresponsiveness, airway caliber and exacerbation rates [20, 48, 59]. The Gaining Optimal Asthma Control study (GOAL) showed that the majority of uncontrolled asthma cases achieved control after one year of treatment using ICS + LABA combination [21].

For the patients with poorly controlled asthma at step 3 and smokers with uncontrolled asthma, the preferred strategy is the addition of a LABA to the medium-dose of ICS, instead of the use of high-dose alone. In the GOAL study, the greatest risk factor for unsuccessful asthma control was permanence of smoking [21, 60].

Spears et al [44] theorize that smokers with asthma shift their dose-response curve showing clinical response only at higher doses of ICS. According to that perspective, Tomlinson et al (2005) published that smokers with mild persistent asthma are insensitive to the therapeutic effect of low dose ICS treatment compared with nonsmokers. The use of higher doses of ICS reduces such disparity in the response between smokers and nonsmokers [48]. In spite of being the only report on this issue of ICS doses in this group of smoking asthmatics, it was considered as a key point on a recent review addressing the challenges of the smoking asthma phenotype [4]. However, additional studies are necessary to confirm those findings and to evaluate the increased risks for long-term adverse effects from higher doses of corticosteroids [15].

### Anti-leukotrienes

The increased synthesis of leukotrienes related to smoking was the basis for testing the leukotriene antagonist montelukast in smokers and non-smokers with asthma, comparing with low doses of ICS. The study showed a small but significant increase in peak expiratory flow in smokers with asthma [19]. Price et al [61] found similar results with the use of the same drug or a high-dose of fluticasone, both compared with placebo. The studied outcomes were the number of days with controlled asthma and improvements in symptom scores. The subjects that used only fluticasone showed significant improvement in FEV1. This result was not detected with montelukast-treated group. However, when the smoking load was controlled, the authors showed that such variable interfered in the results. Patients with a smoking history of less than 11 pack-years exhibited better benefits from fluticasone; individuals with a smoking history of more than 11 pack-years experienced better benefits with montelukast. The explanation of these finding was that the more intensive exposure to tobacco smoke induced an increased synthesis of leukotriene. This group of asthmatic smokers would benefit more from the use of an anti-leukotriene drug (montelukast) to control their chronic smoking asthma than from fluticasone. Subjects with a less-intensive smoking exposure would clinically improve with ICS [61].

This group of drug was included in the GINA 2015 Report for use at step 2 and 3 [58].

### Corticosteroids as extra-fine particles

There are some data on asthma phenotypes in which dysfunction in both the central and distal airways may occur. Small airways seem to be affected more in certain phenotypes, as nocturnal asthma, severe steroid-dependent or difficult-to-treat asthma, asthma complicated by smoking, elderly asthmatic patients and/or patients with fixed airflow obstruction. In these cases, the use of ICS in extra-fine particles with higher deposition rates on the periphery of the airways may be more effective than the use of traditional ICS [62].

This possibility was tested associating extra-fine particles beclomethasone/formoterol in asthmatic smokers. In a real-life scenario, after one year of observation, an improvement in FEV1 and better asthma control was detected, suggesting another possibly effective strategy for the treatment of smokers with asthma. Other studies have shown that smokers with asthma have lower exacerbation rates and are more likely to have controlled asthma with the use of beclomethasone extra-fine particles compared with fluticasone [63, 64]. Ciclesonide extra-fine particles are also being tested for refractory eosinophilic asthma, but not yet, as far as we could determine, with asthmatic smoker.

Using drugs with different mechanisms of action could result in additive effects in reversing resistance to corticosteroids [62]. The role of ICS in extra-fine particles + LABA associated with anti-leukotrienes in asthmatic smokers has not been published.

Another possibility to reverse the non-response to corticosteroids may be theophylline use in low doses. This finding likely arises thanks to the increased activity of HDAC, which is suppressed in smokers [65].

### Other treatments

The possible roles of various other drugs with reasonable theoretical bases in the reversal of the sensitivity of corticosteroids are under studies in smokers with asthma, but these works are still in their initial phase [56]. The use of oral vitamin D3, statins and macrolides are examples, but their clinical efficacy had not yet been proven [8, 66]. In addition, the effect of tobacco smoking on arachidonic acid metabolites in asthmatic subjects seems to be different from that in never-smokers with asthma, a finding that suggests an important pathway to optimize therapeutic responses in the future [67].

The newly introduced drugs in the therapeutic arsenal for treating asthma (indacaterol, an ultra-LABA, and the combination of fluticasone furoate/vilanterol) have not yet been tested in smokers with asthma.

The role of tiotropium in asthma treatment is under investigation, but the majority of the studies exclude current smokers. However, subgroup analyses showed no differences in several outcomes, including smoking history [68].

## Conclusions

1. Smoking is associated with a higher incidence of asthma and asthmatic smokers likely represent a distinct phenotype of the disease. Active smoking and exposure to environmental tobacco smoke in adult life are associated with symptoms suggestive of asthma and with bronchial hyperreactivity.
2. Asthmatic smokers present various worse outcomes compared with non-smoking asthmatics.
3. Smoking cessation improves several asthma outcomes. The respiratory health benefits of not starting smoking and smoking cessation among those who do smoke are of considerable benefit.
4. Smokers with asthma have a lower response to the beneficial effects of corticosteroids, both inhaled (ICS) and orally administered. The ICS combination with LABA induces better response than the isolated use of ICS, even at high doses.
5. All physicians should warn their asthmatic patients about the additional risks related to smoking and help them treat smoking and nicotine addiction. There are special strategies to approach this subgroup of asthmatics.
6. There is an unmet need to improve smoking cessation schemes for asthmatic subjects. New strategies are necessary to assist subjects who cannot or do not wish to stop smoking. Considering that there will be an improvement in the responsiveness to corticosteroids if such patients quit tobacco use, such strategy should be fully explained to the smoking asthmatics. Stopping smoking and the use of corticosteroids will antagonize the pro-inflammatory effects and oxidative stress, which are relevant mechanisms of the disease in smokers with asthma.
7. A new perspective is electronic cigarettes, but many uncertainties currently exist about their utility in smoking cessation. Several new drugs are being introduced in the arsenal of asthma treatment, but most of them have not been teste for the asthmatic smokers.
8. As the Surgeon General stated in the 2014 Report: The clinical implications are clear: people with asthma should not smoke.
